# Report from the 21st Annual Western Canadian Gastrointestinal Cancer Consensus Conference; Calgary, Alberta; 20–21 September 2019

**DOI:** 10.3390/curroncol28050310

**Published:** 2021-09-21

**Authors:** Richard Lee-Ying, Osama Ahmed, Shahid Ahmed, Shahida Ahmed, Oliver F. Bathe, Bryan Brunet, Laura Dawson, Janine Davies, Valerie Gordon, Pamela Hebbard, Jessica Kasnik, Christina A. Kim, Duc Le, Michael K. C. Lee, Howard Lim, John Paul McGhie, Karen Mulder, Jason Park, Daniel Renouf, Vincent Tam, Robin Visser, Ralph P. W. Wong, Adnan Zaidi, Corinne Doll

**Affiliations:** 1 Tom Baker Cancer Center, Alberta Health Service, Calgary, AB T2N 4N2, Canada; Vincent.tam@ahs.ca (V.T.); Corinne.Doll@albertahealthservices.ca (C.D.); 2 Saskatoon Cancer Center, Saskatchewan Cancer Agency, Saskatoon, SK S7N 4H4, Canada; Osama.Ahmed@saskcancer.ca (O.A.); Shahid.Ahmed@saskcancer.ca (S.A.); Bryan.Brunet@saskcancer.ca (B.B.); Duc.le@saskcancer.ca (D.L.); adnan.zaidi@saskcancer.ca (A.Z.); 3 Department of Oncology, Cancer Care Manitoba, Winnipeg, MB R3E 0V9, Canada; Shahida.Ahmed@saskcancer.ca (S.A.); vgordon1@cancercare.mb.ca (V.G.); Pamela.Hebbard@cancercare.mb.ca (P.H.); Ckim3@cancercare.mb.ca (C.A.K.); JPARK@sbgh.mb.ca (J.P.); ralph.wong@cancercare.mb.ca (R.P.W.W.); 4 Surgical Oncology, Arnie Charbonneau Cancer Institute, Calgary, AB T2N 4Z6, Canada; Oliver.Bathe@albertahealthservices.ca; 5 Princess Margaret Cancer Centre, University Health Network, Toronto, ON M5G 2C1, Canada; Laura.Dawson@rmp.uhn.ca; 6 Department of Oncology, British Columbia Cancer Agency, Vancouver, BC V5Z 4E6, Canada; jan.davies@bccancer.bc.ca (J.D.); MichaelKC.Lee@petermac.org (M.K.C.L.); hlim@bccancer.bc.ca (H.L.); drenouf@bccancer.bc.ca (D.R.); 7 Cross Cancer Institute, Alberta Health Services, Edmonton, AB T5G 1Z2, Canada; jessica.kasnik@ahs.ca (J.K.); Karen.Mulder@albertahealthservices.ca (K.M.); 8 Department of Oncology, British Columbia Cancer Agency, Victoria, BC V8R 4S1, Canada; jmcghie@bccancer.bc.ca; 9 Department of Surgery, University of Manitoba, Winnipeg, MB R3E 0V9, Canada; rvisser@exchange.hsc.mb.ca

**Keywords:** hepatocellular carcinoma, stereotactic body radiation therapy, pancreatic cancer, biliary tract cancer, cholangiocarcinoma, adjuvant chemotherapy, surgery

## Abstract

The 21st annual Western Canadian Gastrointestinal Cancer Consensus Conference (WCGCCC) was held in Calgary, Alberta, 20–21 September 2019. The WCGCCC is an interactive multi-disciplinary conference attended by health care professionals from across Western Canada (British Columbia, Alberta, Saskatchewan, and Manitoba) involved in the care of patients with gastrointestinal cancer. Surgical, medical, and radiation oncologists, pathologists, radiologists, and allied health care professionals such as dietitians and nurses participated in presentation and discussion sessions to develop the recommendations presented here. This consensus statement addresses current issues in the management of hepato-pancreato-biliary (HPB) cancers.

## 1. Terms of Reference

### 1.1. Purpose

The Western Canadian Gastrointestinal Cancer Consensus Conference (WCGCCC) aims to develop consensus opinion of oncologists and allied health professionals from across Western Canada, attempting to define best care practices and to improve care and outcomes for patients with gastrointestinal cancers.

### 1.2. Participants

The WCGCCC welcomes medical oncologists, radiation oncologists, surgical oncologists, pathologists, radiologists, gastroenterologists, and allied health professionals from western Canada who are involved in the care of patients with gastrointestinal malignancies (Table 1).

### 1.3. Target Audience

The recommendations presented here are targeted at health care professionals involved in the care of patients with hepato-pancreato-biliary (HPB) cancers.

### 1.4. Basis of Recommendations

The recommendations are based on the presentation and discussion of the best available evidence. Where applicable, references are cited. Additional evidence has been published since the timing of the 2019 meeting, and the authors have incorporated this, where relevant, into each topic discussion. Barriers to access (e.g., approval but no funding) and limitations in data interpretation continue to make the consensus recommendations relevant, even in light of new data.

## 2. Question 1

What is the current role of stereotactic body radiation (SBRT) in hepatocellular carcinoma (HCC)?

### 2.1. Recommendations

The role of SBRT in HCC is evolving and clinical trials should always be considered. Cases should be reviewed in multi-disciplinary rounds. SBRT is an option to be considered alongside other ablative therapies. SBRT as a bridge or downstaging to transplant could also be considered in addition to other local regional strategies.

### 2.2. Summary of Evidence

The role of SBRT continues to be refined, with clinical trial enrollment as the optimal means of advancing its use in HCC. When considered alongside other ablative therapies in a multi-disciplinary discussion, SBRT is best suited for patients with HCC at a higher risk of complications or recurrence following interventional ablative therapies (e.g., tumors >3–5 cm, infiltrative tumors, located near the dome of the liver) [1]. SBRT can also serve as a bridge to or as downsizing for transplant in addition to other local regional strategies. Risks are higher in patients who receive SBRT for central lesions and who go on to have a live donor liver transplant. Thus, multi-disciplinary decision making is crucial for these patients.

Vascular invasion is a poor prognostic factor in HCC patients. Survival is worse in patients with more extensive HCC with vascular involvement (main vessel involvement, occlusive tumor thrombus). For patients with vascular invasion (hepatic vein, portal vein, IVC or branch invasion), SBRT may be used with the goal of debulking or ablating intra-vascular tumor and increasing the chance of recanalization. A randomized phase II study from Korea demonstrated improved progression free survival (PFS) and overall survival (OS) with radiation therapy combined with TACE compared to sorafenib alone, in patients with macrovascular HCC invasion [2].

## 3. Question 2

What is the optimal sequence of systemic therapy in patients with advanced HCC? In the first line setting? In the second line setting?

### 3.1. Recommendations

- Sorafenib or lenvatinib are options for first line therapy in patients who are not eligible for local-regional strategies and have a Child-Pugh A score. Sorafenib can also be considered in Child-Pugh B7 in the absence of ascites. The alternate treatment can be considered in cases of intolerance.
- In the second line setting, options include regorafenib and cabozantinib.
- Clinical trials should be considered. The role of immunotherapy is evolving.

### 3.2. Summary of Evidence

Sorafenib has been the standard treatment for advanced HCC for over a decade since the SHARP and Asia-Pacific trials showed that sorafenib improved OS compared to placebo [3,4]. In 2018, the REFLECT trial showed that lenvatinib is non-inferior for OS when compared to sorafenib in the first-line treatment of advanced HCC [5]. Response rates and PFS favored lenvatinib. The patients included in these trials had Child-Pugh A liver function. For patients with Child-Pugh B liver function, sorafenib appears to be safe according to the GIDEON observational study [6]. Many oncologists would treat patients with Child-Pugh B7 liver dysfunction in the absence of ascites if the patient has a good performance status.

In clinical trials, sorafenib has been shown to be associated with toxicities such as palmar-plantar erythrodysesthesia, diarrhea, hypertension, anorexia, weight loss and fatigue [3,4]. Lenvatinib appears to have lower rates of palmar-plantar erythrodysesthesia, but higher rates of hypertension [5]. Thus, in patients intolerant of sorafenib for such toxicities as palmar-plantar erythrodysesthesia, lenvatinib should be considered. Alternatively, in patients with poorly controlled hypertension, sorafenib would be preferred. Other first-line treatment options, such as the combination of atezolizumab and bevacizumab were not available at the time of this discussion.

In second line setting, post-sorafenib, the RESORCE trial found that regorafenib improves OS compared to placebo (median 10.6 vs. 7.8 months, HR 0.63, 95% CI 0.50–0.79, *p* < 0.0001). Patients in this study must have tolerated and progressed on sorafenib [7]. In 2018, the CELESTIAL trial found that cabozantinib improves OS compared to placebo (median 10.2 vs. 8.0 months, HR 0.76 95% CI 0.63–0.92, *p* = 0.005) when given to patients who have been treated with up to two lines of systemic therapy for hepatocellular carcinoma, including previous treatment with sorafenib [8]. The REACH-2 trial evaluated patients after first-line sorafenib with a poorer prognosis with an AFP ≥400 ng/mL, and found that ramucirumab led to a more modest improvement in OS compared to placebo (median 8.5 vs. 7.3 months, HR 0.710, 95% CI 0.531–0.949, *p* = 0.0199), which though statistically significant may not be as clinically significant [9].

The prognosis of advanced stage HCC is still very poor despite the recent advances in systemic treatment. Immunotherapy drugs are currently being studied in clinical trials. However, two reported phase III trials of anti-PD1 drug monotherapy (nivolumab, pembrolizumab) have failed to show a statistically significant improvement in overall survival in first- and second-line treatment, respectively, despite previous accelerated FDA conditional approval [10,11]. Trials of combination treatments including immunotherapy and tyrosine-kinase inhibitors in the first-line setting for advanced HCC are ongoing. The combination of atezolizumab and bevacizumab, compared to sorafenib in the IMbrave150 study, presented at ESMO Asia 2019, after the WCGCCC meeting, suggests that the combination is superior to sorafenib and likely to become a new standard of care in the first line setting [12]. The combination of atezolizumab and bevacizumab is Health Canada approved and received a conditional approval from the national oncology-specific health technology assessment body pan-Canadian Oncology Drug Review (pCODR) assuming the cost-effectiveness of the treatment can be improved upon [13]. Despite support for its use, funding negotiations remain unresolved, limiting its use to a minority of patients who can afford to pay out of pocket or with the assistance of a private drug plan. Patients with a history of autoimmune disease or uncontrolled varices are also ineligible, keeping discussions about lenvatinib and sorafenib in the first-line setting relevant.

Existing second line studies all used sorafenib as a first line treatment, so it is difficult to interpret the efficacy of these novel agents in the context of new data. The RESORCE trial excluded patients who had other systemic treatments beyond sorafenib, while the CELESTIAL trial included patients with two or more systemic therapies, including 17 patients who had prior immunotherapy, though not specifically in the combination of atezolizumab and bevacizumab [7,8]. Further expert opinion and limited real-world studies have suggested that the activity of sorafenib and lenvatinib, post-atezolizumab and bevacizumab may have similar activity to the first line setting, suggesting a simple shift in the sequencing discussed at the 2019 WCGCCC meeting [14,15].

## 4. Question 3

What are the important dietary interventions in the management of pancreatic cancer? What is the role of pancreatic enzyme replacement therapy?

### 4.1. Recommendations

All patients with pancreas cancer should be referred to a registered dietitian for specialized nutritional care due to the high risk of malnutrition. Initial care providers should screen patients for pancreatic enzyme deficiency and initiate replacement if needed.

### 4.2. Summary of the Evidence

Pancreatic cancer is a very aggressive malignancy that is often not diagnosed until an advanced or metastatic stage. More than 80% of patients with PDAC present with malnutrition at diagnosis due to multifactorial causes [16,17]. It has been estimated that 10–20% of deaths in patients with cancer are related to malnutrition rather than their malignancy [18]. Therefore, it is essential to identify, understand the etiology, and apply appropriate nutritional interventions to curtail the deleterious symptoms of malnutrition and improve clinical outcomes. The three factors which expose pancreatic cancer patients to such a high risk of malnutrition are (1) disease-related malnutrition (anorexia, hypermetabolism), (2) treatment side effects (nausea, vomiting, constipation, diarrhea, mouth sores, taste changes), and (3) exocrine/endocrine dysfunction. A consultation with a specialized registered dietitian can support the patient and team in the identification and management of these issues.

In the setting of cancer, both pancreatic exocrine and endocrine functions can be affected. Endocrine cells make up approximately 5% of the pancreas and function to regulate metabolism in the body through the production and secretion of hormones such as insulin and glucagon. Derangements in this system will present as diabetes mellitus [18]. The majority of pancreatic cells, however, are exocrine cells. The pancreatic exocrine cells produce digestive juices (bicarbonate) and enzymes (protease, amylase, and lipase) which are essential for the neutralization of gastric chyme and the digestion of all macronutrients. Pancreatic enzyme insufficiency (PEI) is defined as the reduced or inappropriate secretion or activity of pancreatic juice and its digestive enzymes, particularly pancreatic lipase. The presence of PEI affects a patients’ ability to properly digest and absorb protein (protease), fat (lipase), and complex carbohydrates (amylase).

When assessing this population for PEI, it is important to consider location, size, history of chronic pancreatitis, ductal obstruction and surgical intervention to the pancreas and tumor. The prevalence of PEI post Whipple procedure is 80–90% compared to 20–50% in patients who underwent a distal pancreatectomy. In contrast, 20–60% of patients with unresectable pancreatic adenocarcinoma have PEI [19].

Signs and symptoms of PEI can be broken down into three main categories; abdominal, nutritional and endocrine. Abdominal symptoms include diarrhea, steatorrhea, fecal urgency, bloating, malodorous gas, GERD, cramping and abdominal pain, and post prandial gurgling. Nutritional symptoms include weight loss, sarcopenia, weakness and fatigue, food avoidance, and fat soluble vitamin deficiency. Endocrine symptoms will present with hypoglycemia or decreased hyperglycemic agent requirements. If a patient has pancreatic adenocarcinoma and any of the above symptoms, the intervention recommended is pancreatic enzyme replacement therapy (PERT) [19,20]. Patients who have an intact stomach taking enzymes orally require enterically coated versions. The two main brands of these enzymes used in Canada are Creon 10 and 25 and Cotazym ECS 8 and 20. Non-enterically coated enzymes (Viokase, Cotazym) are required for patients with a feeding tube (gastrically or jejunely) or post-gastrectomy. Each capsule of PERT has a combination of protease, lipase and amylase, as broken down in Table 2.

The dosing of PERT is primarily based on the cystic fibrosis population and additional enzyme replacement is likely needed for the pancreatic cancer population [16]. PERT is prescribed based on per kilogram body weight of the patient, in a range of 500–2500 units of lipase per kilogram body weight per meal, up to 10 000 units of lipase per kilogram of body weight per day. The risk associated with exceeding this is constipation. PERT should be taken before all meals and snacks that contain macronutrients and will last for up to 1 h after consumption. Larger meals or meals that contain a higher amount of fat will require more PERT for adequate digestion.

The European Society for Clinical Nutrition and Metabolism (ESPEN) and the American Society for Parenteral and Enteral Nutrition (ASPEN) guidelines suggest no macronutrient restriction, with protein requirements elevated to 1.2–2.0g/kilogram body weight/day. Patients on appropriate PERT should feel an improvement in symptom burden and will better absorb the nutrients they are ingesting.

## 5. Question 4

What is the optimal neoadjuvant/adjuvant strategy for pancreas cancer?

### 5.1. Recommendations

- Six months of mFOLFIRINOX is standard adjuvant therapy for patients with resected pancreatic cancer. In patients who are not candidates for mFOLFIRINOX, a combination of gemcitabine/capecitabine or gemcitabine alone or 5FU alone can be considered.
- The role of adjuvant radiation is not well defined but may be considered in patients with high risk of local recurrence, in the context of a multidisciplinary discussion.
- Cases should be reviewed in a multi-disciplinary fashion to determine the intent and strategy for borderline resectable pancreas cancer. Clinical trials should be considered for these patients. Neoadjuvant chemotherapy using FOLFIRINOX is the preferred strategy. Gemcitabine and Nab-paclitaxel can be considered depending on patient factors and tolerability. Chemoradiotherapy or radiotherapy could be considered in select cases.
- In resectable cases, there is no evidence-based role for neoadjuvant chemotherapy to date; clinical trials should be considered.

### 5.2. Summary of Evidence

Pancreatic ductal adenocarcinoma (PDAC) is an aggressive cancer, and only approximately 20% of cases present when disease is localized and amenable to surgical resection [21]. Various trials have assessed the impact of adjuvant therapy after surgical resection of PDAC. The ESPAC-1 study was a two-by-two factorial design, randomizing patients to adjuvant chemoradiation, chemotherapy, both treatments or observation alone, with a goal of assessing the impact of chemoradiation versus no chemoradiation, and chemotherapy versus no chemotherapy on two-year OS [22]. Accrual was poor, therefore patients were accrued in a parallel study known as ESPAC-1 plus, where they were randomized to adjuvant chemotherapy with bolus 5-fluorouracil (5FU) and folinic acid versus observation, and clinicians were able to provide chemoradiotherapy if they felt that it was clinically indicated [23]. Overall, the results of ESPAC-1 showed a benefit to adjuvant chemotherapy, with a two-year OS of 40% with chemotherapy compared to 30% with no chemotherapy, and possible harm to the administration of adjuvant chemoradiation, with a two-year OS of 29% with chemoradiation compared to 41% amongst those who received no chemoradiation. ESPAC-1 was not powered to assess the impact of adjuvant chemotherapy to observation, however, the ESPAC-1 plus results showed a similar benefit to adjuvant chemotherapy. The interpretation of these studies is limited by a number of factors, including the complicated study design and radiation techniques used.

However, further trials demonstrated the utility of adjuvant chemotherapy in resectable PDAC. The CONKO-001 trial randomized patients who had undergone a complete macroscopic resection to six cycles of gemcitabine 1000 mg/m^2^ on days 1, 8 and 15 of a 28-day cycle or observation [24,25]. There was a statistically significant improvement in disease free survival (DFS) (median DFS 13.4 months with gemcitabine versus 6.7 months with observation) and OS (5-year OS 20.7% with gemcitabine versus 10.4% with observation), and this benefit persisted in patients with both R0 and R1 resections. A randomized comparison of adjuvant gemcitabine to 5FU showed no difference in OS, however, there was more grade 3 toxicity, including stomatitis, diarrhea and hospitalizations, with 5FU compared to gemcitabine [26].

The ESPAC-4 study addressed whether six months adjuvant gemcitabine and capecitabine, was superior to gemcitabine alone [27]. Patients with macroscopically complete resection of PDAC, with no evidence of metastatic disease, an Eastern Cooperative Group (ECOG) performance score (PS) of 0–2, who had not received any neoadjuvant chemotherapy, were randomized to combination therapy with gemcitabine 1000 mg/m^2^ weekly on days 1, 8 and 15 of a 28 day cycle and capecitabine 1660 mg/m^2^ for 21 days followed by a 7 day break, versus gemcitabine alone. Results were stratified by country of enrollment and resection margin status (R0 versus R1). Treatment was to be started within 12 weeks of surgery and enrollment in ESPAC-4 was not restricted by post-operative CA 19-9 levels. The primary endpoint was OS and secondary endpoints included survival at 24 months and 5 years, relapse free survival (RFS), toxicity and quality of life as measured by the EORTC QLQ-C30 questionnaire. The study cohort included patients with a poor prognosis, as 60% of patients had R1 resection margins, 80% had lymph node positive disease, and post-operative CA 19-9 levels ranged from 0.1 to >8000. The median OS with gemcitabine and capecitabine was 28.0 months compared to 25.5 months with gemcitabine alone (hazard ratio 0.82, 95% CI 0.68–0.98, *p* = 0.032). On multivariable analysis, tumor grade, lymph node status, maximum tumor size, resection margin status and post-operative CA 19-9 levels were independent predictors of OS. Interestingly, there was no difference in RFS between combination therapy and gemcitabine alone (HR 0.86, 95% CI 0.73–1.02, *p* = 0.082). There was increased grades 3 and 4 toxicity with combination therapy, including increased neutropenia, infections, diarrhea and hand foot syndrome.

APACT is a phase III randomized controlled trial assessing adjuvant nab-paclitaxel plus gemcitabine (NG) compared to gemcitabine alone, with a primary endpoint of independently assessed DFS [28]. Patients with resected PDAC with no evidence of metastatic disease and a post-operative CA 19-9 < 100 were randomized in a 1:1 fashion to the two treatment arms. Results were stratified by geographic region, resection margin status and lymph node status. In total, 24% of patients had R1 margins and 72% had lymph node positive disease. There was no difference in independently assessed DFS between the two treatment groups. The median DFS with NG was 19.4 months, compared to 18.8 months with gemcitabine alone (hazard ratio 0.88, 95% CI 0.73–1.06, *p* = 0.18). In a pre-planned subgroup analysis, the groups that appeared to benefit from combination therapy included moderate compared to poorly differentiated tumors, lymph node positive disease and normal CA 19-9. There was a statistically significant difference in investigator-assessed DFS, at 16.6 months for NG, compared to 13.7 months for gemcitabine (hazard ratio 0.82, 95% CI 0.694–0.965, *p* = 0.0168). The difference between independent and investigator assessed DFS may be related to the clinical features that physicians take into consideration, alongside radiologic findings, when assessing disease progression in patients with PDAC. The median OS for those who received NG was 40.5 months, compared to 36.2 months for gemcitabine alone (hazard ratio 0.82, 95% CI 0.68–0.996, *p* = 0.045), although data are not yet mature.

Investigation of a more intensive adjuvant regimen was investigated in the PA.6 trial [29]. Patients with macroscopic resection of PDAC, with no evidence of metastatic disease were randomized to six months of modified FOLFIRINOX (mFOLFIRINOX), consisting of 46 h infusion of 5FU (2400 mg/m^2^), leucovorin (400 mg/m^2^), oxaliplatin (85 mg/m^2^) and irinotecan (150 mg/m^2^) given once every two weeks, or gemcitabine 1000 mg/m^2^ given on days 1, 8 and 15 on a 28 day schedule. Criteria for enrollment in this study were more stringent, as patients had to be <79 years old, have an ECOG PS of 0–1, with a bilirubin level < 1.5 times the upper limit of normal, a creatinine clearance of >50 mL/minute, and a post-operative CA 19-9 level <180. The primary endpoint was DFS and other endpoints included OS and safety. Results were stratified by trial center, nodal status, margin status and post-operative CA 19-9 levels. With this stricter inclusion criteria, approximately 40% of patients had R1 resections, 73% had stage IIB disease, and 0.4% had stage III disease. The median DFS with mFOLFIRINOX was 21.6 months, compared to 12.8 months with gemcitabine (stratified hazard ratio 0.58, 95% CI 0.46–0.73, *p* < 0.001). This benefit with mFOLFIRINOX was seen in all subgroups, including those with T3/4 tumors, N1 disease and R1 resections. In an exploratory subgroup analysis of patients >70 years old, representing 20.5% of the study population, the benefit of mFOLFIRINOX over gemcitabine did not reach significance (hazard ratio 0.86, 95% CI, 0.53–1.39). On multivariable analysis, factors independently predictors of worse DFS included tumor grade and portal vein resection. Median OS was also improved with mFOLFIRINOX at 54.5 months compared to 35.0 months with gemcitabine (stratified hazard ratio for death, 0.64; 95% CI, 0.48–0.86; *p* = 0.003). It should be noted that the median OS seen in the gemcitabine group is longer than what has been seen historically, with many potential reasons, including better surgical technique, patient selection and post-progression therapies. As expected, mFOLFIRINOX was associated with increased toxicity. Grade 3/4 toxicity occurred in 52.9% of patients receiving mFOLFIRINOX, compared to 12.2% of those receiving gemcitabine. Specifically, there was more mucositis, nausea, vomiting, diarrhea, abdominal pain, fatigue, elevated GGT and paresthesias with mFOFLIRINOX. Over 60% of those receiving mFOLFIRINOX received growth factor support, compared to 3.7% of those receiving gemcitabine. There was no difference in grade 3/4 toxicity based on age <70 or >70. Due to the superior survival seen with mFOLFIRINOX, it is the adjuvant chemotherapy regimen of choice; however, factors such as age, ECOG PS, comorbidities, post-operative CA 19-9 and chemotherapy toxicity profile should be taken into consideration.

The role of adjuvant radiation is not well defined and results from randomized trials are conflicting. Overall, there is no strong evidence for using radiation therapy for a survival improvement. Adjuvant radiation therapy may be considered in selected patients, following systemic therapy, with the primary goal of reducing the risk of local-regional recurrence and maintaining quality of life. In resectable cases, there is no evidence for the routine use of neoadjuvant radiation therapy [30]. When radiation therapy is used for the treatment of pancreatic cancer, pre-radiation treatment anti-emetics are recommended. For patients with tumors close to the stomach or duodenum (the great majority of pancreatic cancers), proton pump inhibitors are recommended to reduce the risk of gastric toxicity [30].

When deciding on whether a neoadjuvant approach or an upfront surgical approach should be taken, it is critical to clearly define whether a PDAC is resectable, borderline resectable, borderline unresectable or unresectable. Definitions of resectability have evolved over time and require sufficient radiographic and surgical expertise to determine. The nature and extent of vascular involvement is often a primary determinant of resectability, with abutment indicating ≤180 degree involvement and encasement >180 degree involvement of a particular vessel. To be considered borderline resectable, most guidelines permit superior mesenteric vein/portal vein encasement, as long as reconstruction of the vessel is feasible, while the superior mesenteric artery can typically only have abutment for a case to be considered borderline resectable [31,32,33,34,35,36]. Borderline resectable cases typically permit common hepatic artery abutment or short-segment encasement, while most guidelines indicate celiac artery abutment or encasement would render pancreatic cancer unresectable [31,32,33,34,35,36]. Notably, there can be exceptions to arterial involvement, as arterial replacement or resections can occasionally be performed by an experienced hepatobiliary surgeon. Given the complexity of the determination, it often falls to the hepatobiliary surgeon with or without a multidisciplinary discussion to elucidate what is safe and feasible. Clear communication is important to set patient and provider expectations, particularly when disease is either unresectable or only borderline resectable so that management plans can be appropriately aligned with realistic goals.

The role of neoadjuvant chemotherapy in patients with resectable disease is unclear. Offering chemotherapy up front in patients with disease that is amenable to surgical resection may offer some benefits. Neoadjuvant treatment may allow for an assessment of tumor biology, including an evaluation of whether disease responds to aggressive chemotherapy. Those with progressive disease despite optimal chemotherapy may be spared from an aggressive and potentially morbid surgery. Giving neoadjuvant chemotherapy has the potential to treat micrometastatic disease and increase R0 resections. Due to the potential for post-operative complications, which may delay or limit the ability to administer adjuvant chemotherapy, giving chemotherapy before surgery may be preferred. Despite these potential benefits, the role of neoadjuvant chemotherapy in patients with resectable PDAC remains unclear, as there is a lack of evidence to support such an approach. A systematic review and meta-analysis attempted to address this question [37]. When including only randomized and prospective phase II and III trials comparing neoadjuvant to adjuvant therapy, only two phase II papers, one of which was randomized, were identified. The inclusion criteria were therefore expanded to include an additional four prospective and three retrospective studies. The analysis favored neoadjuvant therapy for increasing R0 resections and survival at various time points, however, the poor quality and small number of studies limits the ability to make strong conclusions about the role of neoadjuvant therapy in this setting, and ultimately, upfront surgery followed by adjuvant chemotherapy remains the standard of care for patients with resectable PDAC. Ongoing trials, such as the NEOPAC (NCT01521702) and ALLIANCE A021806 studies, will add further information about the role of neoadjuvant therapy for PDAC. One of the challenges of trials in this setting will be to ensure clear definitions of resectable disease and confirming trial participants are indeed eligible [38], and the endpoints chosen in trials should reflect clinically relevant endpoints including OS and R0 resection rates.

The role of neoadjuvant chemotherapy in borderline resectable PDAC is also unclear. In a phase 2 study of 48 patients with borderline resectable PDAC determined by a multidisciplinary team review, patients received pre-operative FOLFIRINOX followed by neoadjuvant chemoradiation [39]. The primary outcome was R0 resection rate, which was achieved in 65% of patients. The median PFS was 14.7 months and the median OS was 37.7 months. A meta-analysis compared the impact of neoadjuvant therapy (chemotherapy, chemoradiation or both) versus upfront surgery on survival in patients with resectable or borderline resectable PDAC [40]. The median OS with any neoadjuvant therapy was 18.8 months, compared to 14.8 months for those who underwent upfront surgery. A retrospective study of patients with borderline resectable and locally advanced PDAC receiving neoadjuvant FOLFIRINOX versus upfront surgery showed that 92% of patients had an R0 resection who received neoadjuvant FOLFIRINOX [41]. Given the heterogeneity of the populations included in these studies (resectable, borderline resectable and locally advanced disease), as well as the heterogeneity of the neoadjuvant therapies used, one should be cautious when interpreting these results. Ultimately, clear definitions of resectability and well-designed randomized phase 3 trials are required to answer the question of the role of neoadjuvant chemotherapy, and the optimal regimen, in borderline resectable PDAC.

Currently, if patients are to receive neoadjuvant therapy for PDAC, this would best be done on a clinical trial. Cases should be reviewed in a multi-disciplinary fashion to determine the intent and strategy for borderline resectable pancreas cancer as it is important to set realistic expectations for patient outcomes. Neoadjuvant chemotherapy using FOLFIRINOX is the preferred strategy due to its objective response rate (31.6%) in the metastatic setting and efficacy in the adjuvant setting [29,42]. Notably, an objective response may not translate to conversion from borderline resectable to resectable disease, as there may be specific anatomic considerations required for a response to allow for surgical resectability. NG can be considered depending on patient factors and tolerability, though it does have a lower objective response rate (23%) in the metastatic setting and more questionable benefit in the adjuvant setting [28,43]. Chemoradiotherapy or radiotherapy alone may be considered in selected cases following systemic therapy. The primary role of radiation therapy is to increase the chance of a R0 resection margin and to reduce the risk of local recurrence. Capecitabine is the preferred radiation sensitizer for patients who have previously received FOLFIRINOX, and it has been shown to be associated with less toxicity than gemcitabine-based chemo-radiation therapy [44]. Gemcitabine-based chemo-radiation therapy is an alternative for patients who have not received FORFIRINOX.

Published after the WCGCCC meeting, the PREOPANC trial randomized patients with resectable and borderline resectable pancreatic cancer to receive chemoradiation with gemcitabine and surgery or surgery alone, followed by adjuvant gemcitabine [45]. This trial demonstrated an improvement in surrogate endpoints such as R0 resection rates (71 vs. 40%), disease-free survival and locoregional failure-free interval with the use of neoadjuvant chemoradiation. No OS benefit was observed initially, though with longer follow-up a potential survival benefit has been suggested [45,46]. However, this trial utilizes inferior systemic treatment in the control arm with gemcitabine alone and may not be reflective of modern practice. The phase II SWOG S1505 study compared neoadjvuant treatment with mFOLFIRINOX and nab-pacltaxel/gemcitabine and found similar R0 resection rates (73 vs. 70%) but failed to meet pre-specified endpoints to be carried forward [47]. The results from further trials such as PREOPANC II, using FOLFIRINOX are awaited [48]. Despite new data since the WCGCCC meeting, routine use of neoadjuvant treatment remains an area of active research, and there remains a need for better powered studies to determine who best to adopt a neoadjvuant approach for.

## 6. Question 5

What are the current standard surgical options for patients with hepatocellular carcinoma?

### 6.1. Recommendations

- Patients should be referred to a hepatobiliary surgeon to review local regional strategies and for multi-disciplinary review. Multi-disciplinary review should involve representatives from transplant, surgery, radiation oncology, medical oncology and interventional radiology. Surgery remains the gold standard for resectable HCC if able to obtain an adequate future liver remnant (FLR). Transplantation is preferred for non-resectable HCC patients with cirrhosis within transplant criteria.

### 6.2. Summary of Evidence

Over recent years there have been significant technological and scientific developments related to the treatment of hepatocellular carcinoma. As a result, there are more locoregional treatment options as well as systemic treatment options. Locoregional treatment options include resection, including laparoscopic resection, liver transplantation, thermal ablation (including radiofrequency ablation and microwave ablation), stereotactic body radiotherapy (SBRT), transarterial chemoembolization (TACE), brachytherapy (where available), and transarterial radioembolization (TARE). The nuances of each of these therapeutic modalities, including case-specific feasibility, limitations and precautions, require expert input from specialists from different disciplines. Therefore, an effective multidisciplinary discussion requires the involvement of surgeons, hepatologists, interventional radiologists, radiation oncologists and medical oncologists, as well as diagnostic radiologists.

The diagnosis of HCC is often made based on imaging, without a biopsy. The Liver Imaging Reporting and Data System (LI-RAD) classification of liver lesions enables estimation of the likelihood that a liver lesion is a hepatocellular carcinoma [49]. Expert assessment of liver lesions by a radiologist is therefore integral to clinical management.

Decisions related to the optimal management of hepatocellular carcinoma require a full appreciation of the patient’s comorbidities and performance status, as well as an assessment for underlying liver disease and portal hypertension. A full medical evaluation is essential. Liver function is first evaluated by applying Child-Pugh criteria [50], which requires INR, albumin, total bilirubin, and evaluation for ascites and encephalopathy. If transplantation is being considered, the MELD score can be calculated (based on creatinine, bilirubin, INR and serum sodium) [51]. Good renal function is essential for TACE. If it is unclear whether hepatic fibrosis or cirrhosis is present, ultrasound elastography or MR elastography is helpful [52]. The presence of portal hypertension can be deduced if varices are seen on cross-sectional imaging or esophagogastroscopy, or if there is evidence of hypersplenism such as a large spleen or thrombocytopenia.

Anatomic factors must also be considered. The intent of resection is curative and therefore, it must be feasible to remove the entirety of the tumor while leaving sufficient liver remnant to avoid liver failure. Occasionally, it may be necessary to perform a portal vein embolization on the side that is being removed in order to induce hepatic hypertrophy on the liver remnant. Thermal ablation should be avoided in lesions located centrally, near the bifurcation of the porta hepatis; lesions located near large vessels are susceptible to heat sink [53], limiting the effectiveness of the procedure; accessing lesions at the liver surface should consider the approach due potential risks of tumor seeding. While transplantation is the best option for healthy patients with poor liver function, it is not indicated in the presence of a large tumor burden. The Milan criteria (a solitary lesion ≤5 cm maximal diameter or up to 3 lesions ≤3 cm) [54] represent conservative criteria, but extended criteria have been more recently introduced with acceptable results [55,56].

The Barcelona Clinic Liver Cancer (BCLC) staging system represents one construct that can help to guide treatment options [57]. The BCLC staging system takes into account the Child-Pugh grade of liver function, the size and number of nodules, and performance status (Table 3). In addition, the platelet count should be considered in treatment decisions. The optimal candidate for resection is an individual who is in good medical condition, has good performance status, has good hepatic functional reserve (Child-Pugh A), has a normal platelet count and has no varices. This corresponds to patients in BCLC Stage 0 or A. Having said that, as described above, there are many nuances to decision-making, and alternatives to resection could also be considered. While the BCLC staging system is an excellent construct to help make treatment decisions, it must be appreciated that there are numerous treatment options for each BCLC stage. The best options are a product of local expertise, and they will evolve as the clinical science improves.

## 7. Question 6

What is the standard second line treatment option in patients with advanced pancreatic cancer? What is the role of targeted treatments for advanced pancreatic cancer?

### 7.1. Recommendations

- Post-FOLFIRINOX, second line gemcitabine + nab-paclitaxel is the preferred regimen. Gemcitabine alone can also be considered in patients not able to tolerate the combination.
- Post nab-paclitaxel + gemcitabine, nanoliposomal irinotecan + 5FU or OFF are preferred regimens.
- For MSI-high, MMR-deficient pancreatic cancer, pembrolizumab should be considered for chemotherapy refractory disease. The role of next generation sequencing is experimental and targeted therapies are not routinely recommended outside of a clinical trial.

### 7.2. Summary of Evidence

The standard first line therapy options for patients with metastatic pancreatic ductal adenocarcinoma (mPDAC) with good performance status include FOLFIRINOX and gemcitabine and nab-paclitaxel [42,43]. The choice between the two regimens is often dependent on the patient’s co-morbidities and physician/patient preference. Although survival outcomes are inferior with gemcitabine alone, it has demonstrated symptomatic clinical benefit, and can be considered when combination therapy is not feasible [58].

For patients who have progressed from FOLFIRINOX, there is no standard chemotherapy proven with phase III evidence. However, inferring from retrospective studies (ranging from 7 to 69 patients), gemcitabine with nab-paclitaxel has an overall response rate (ORR) of approximately 18% with a median PFS of 3 months and median OS of 5–8 months from the date of starting second line therapy [59,60,61,62,63,64,65,66]. Given the regimen appears efficacious in the second line, it is the preferred regimen for patients who can tolerate combination chemotherapy. For those that cannot, gemcitabine monotherapy may be considered [59].

For patients who have progressed on gemcitabine and nab-paclitaxel, liposomal irinotecan and fluorouracil as per NAPOLI-1 trial and OFF regimen as per CONKO-3 trial are reasonable treatment options with phase III evidence [67,68]. Of note, the PANCREOX study showed conflicting results with the mFOLFOX6 regimen having inferior mOS compared to infusional 5FU (6.1 vs. 9.9 months, HR 1.78 (95%CI 1.08–2.93), *p* = 0.024) [69]. Therefore, if an oxaliplatin based regimen is utilized then oxaliplatin should be given as per the OFF regimen.

For patients with mPDAC who have known germline BRCA mutations or DNA damage repair (DDR) genes then second line treatment with platinum-based chemotherapy may be considered, especially if they are platinum naïve. This is supported by the Know Your Tumour Type study, where patients with advanced PDAC with DDR (n = 54) was predictive of significant improvement in mOS if treated with platinum-based therapy (2.37 vs. 1.45 years, *p* < 0.0001) [70]. Similarly, in a smaller study of 71 patients with somatic BRCA mutations where those treated with platinum (*n* = 22) versus not (*n* = 21), mOS was improved to 22 vs. 9 months, *p* = 0.03915 [71]. The DDR genes of interest are pathogenic alterations in BRCA1/2, PALB2, ATM, ATR, ATRX, BAP1, BARD1, BRIP1, CHEK1/2, RAD50/51/51B, or FANCA/C/D2/E/F/G/L if this prospective trial evidence is to be applied. Of note, patients with DDR aberrations may also be more sensitive to 5-FU and irinotecan [72]. For liposomal irinotecan, the benefit seems to be in irinotecan naïve patients where a retrospective study by Glassman DC et al. showed reduced ORR and mOS in prior irinotecan treated patients and naïve patients had similar benefit as per the NAPOLI-1 trial [73].

Beyond chemotherapy, emerging actionable targets with survival benefits are being identified through next generation sequencing (NGS), particularly in patients with KRAS wildtype mPDAC [74]. However, targeted therapies are not routinely considered outside of a clinical trial. In 0.5–0.8% mPDAC patients with deficiency in mismatch repair protein (dMMR) or microsatellite instability (MSI-H), pembrolizumab has shown to be efficacious [75,76,77]. NTRK fusion inhibitors with larorectinib and entrectinib have been recognized as efficacious across multiple tumor types including mPDAC for patients who harbor the fusion [78,79]. NRG1 is another recurrent fusion found across multiple tumor types with a prevalence of approximately 0.3% in mPDAC [80]. There is emerging evidence that it can be targeted with afatinib with a quick and durable response [81,82]. The logistical challenge in reliable detection of such a rare mutation across the population poses a barrier to its utility in today’s clinical practice.

There is recognition of 5-10% hereditary cancer risk associated with mPDAC diagnosis, which may be higher in certain subpopulations [83,84,85]. ASCO and NCCN guidelines recommend that germline testing be considered for all patients with mPDAC in recognition that up to half of the patients may not have a classical family history and germline BRCA mutations may carry treatment implications [86,87,88,89]. However, the implementation of testing across the population remains a significant barrier and is subject to ongoing research [89]. After an initial response to platinum-based chemotherapy, the use of maintenance olaparib compared to placebo demonstrated improved PFS, suggesting activity with the parp inhibitor, but the lack of OS improvement and use of placebo as a control (as opposed to ongoing chemotherapy) casts doubt over its utility despite FDA approval [85].

## 8. Question 7

What is the preferred adjuvant therapy for patients with early stage biliary tract cancer? What are the preferred first- and second-line systemic therapies in patients with advanced biliary tract cancer? What are potential targeted treatments for biliary tract cancer?

### 8.1. Recommendations

- In the adjuvant setting, capecitabine for 6 months is the preferred option. For positive margin disease, patients should be reviewed in a multi-disciplinary fashion to determine if radiotherapy is reasonable.
- In patients with advanced biliary tract cancers, the preferred first line option is gemcitabine and cisplatin. Gemcitabine is an option in patients who cannot tolerate combination therapy.
- Fluoropyrimidine-based chemotherapy may be considered in the second line setting.
- The role of molecular testing and targeted therapy is evolving. For MSI-high, MMR-deficient advanced biliary cancer, pembrolizumab should be considered for chemotherapy refractory disease.

### 8.2. Summary of Evidence

The phase III BILCAP trial, randomized 447 patients with resected biliary tract cancers to receive eight cycles of capecitabine (1250 mg/m^2^ twice daily on days 1–14 every 21 days) or placebo [90]. In total, 38% had R1 resection. The primary intention-to-treat analysis for OS did not meet statistical significance (51.1 vs. 36.4 months, HR 0.81 95% CI 0.63–1.04, *p* = 0.097), while per-protocol analysis did (53 vs. 36 months, HR 0.75, 95% CI 0.58–0.97, *p* = 0.028). Additionally, pre-planned sensitivity analyses adjusting for nodal status, disease grade and sex suggested that capecitabine was beneficial (HR 0.71, 95% CI 0.55–0.92, *p* = 0.010) and RFS was improved in both intention to treat and per-protocol analysis. Given the magnitude of benefit and supportive analyses, adjuvant capecitabine has been adopted as a new standard of care for patients with resected biliary tract cancer. The benefit of adjuvant radiation or chemoradiation is unclear as there is no prospective, randomized data to support its routine use. However, retrospective data suggests potential benefit, particularly in patients with positive margins, so it is reasonable to have these cases reviewed in a multidisciplinary fashion to determine if radiotherapy should be considered [91].

Cisplatin and gemcitabine are recommended for first line treatment of metastatic cholangiocarcinoma based on the multicenter ABC-02 trial. In this study, 410 patients were randomly assigned to eight cycles of cisplatin (25 mg/m^2^) followed by gemcitabine (1000 mg/m^2^) on days 1 and 8 every 21 days, or gemcitabine alone (1000 mg/m^2^ on days 1, 8, and 15 every 28 days) [92]. Median OS was significantly greater with combination therapy (11.7 versus 8.1 months), as was median PFS (8 versus 5 months). For second-line, ABC-06 was the first prospective phase III trial evaluating the benefit of chemotherapy after Cisplatin and Gemcitabine in patients with advanced cholangiocarcinoma. The trial compared infusional fluorouracil plus leucovorin and oxaliplatin (FOLFOX) with active symptom control. FOLFOX improved OS at 6 (61 versus 36%) and 12 months (26 versus 11%) [93]. The incremental benefit of oxaliplatin in addition to fluoropyrimidine alone after cisplatin and gemcitabine is unclear. Whilst there is a lack of prospective evidence, retrospective comparisons indicate oxaliplatin combination achieves a higher objective response rate (8 vs. 1%, *p* = 0.009), but PFS and OS are not clearly different from a fluoropyrimidine alone [94].

Molecular targeted therapy has been an emerging area of interest for biliary tract cancers. As with other cancers that are dMMR/MSI-H, immunotherapy has demonstrated activity for this disease subtype [74,95]. Similarly, TRK inhibitors like larorectinib and entrectinib have been recognized as efficacious for TRK-fusion positive cholangiocarcinomas as well [77,78]. Several trials are also evaluating the role of FGFR2 transloactions, which are present in approximately 13% of patients with cholangiocarcinoma [96]. Pemigatinib has subsequently received FDA approval due to an objective response rate of 36% and disease control rate of 80% [97]. Infigratinib is another FGFR2 targeted therapy that has received accelerated approval by the FDA [98]. The emerging number of targeted therapies available for intrahepatic cholangiocarcinomas has led to a blanket recommendation by ESMO for routine next generation sequencing for these cancers [99]. However, funding for such testing remains elusive in the publicly funded context of Canada.

## Figures and Tables

**Table 1:** List of 2019 WCGCCC participants.

Prefix	First Name	Last Name	Job Title	Organization
Dr.	Shahid	Ahmed	Medical Oncologist	Saskatchewan Cancer Agency
Dr.	Osama	Ahmed	Medical Oncologist	Saskatoon Cancer Centre
Dr.	Oliver	Bathe	Professor	University of Calgary
Dr.	Malcolm	Brigden	Medical Oncologist	Alberta Health Services
Dr.	Bryan	Brunet	Radiation Oncologist	Saskatoon Cancer Center
Dr.	Julianna	Caon	Radiation Oncologist	BC Cancer
Dr.	Haji	Chalchal	Medical Oncologist	Allan Blair Cancer Ctr
Dr.	Janine	Davies	Medical Oncologist	BC Cancer
Dr.	Laura	Dawson	Professor	Princess Margaret Cancer Centre
Dr.	Sonny	Dhalla	Surgeon	Brandon
Dr.	Corinne	Doll	Radiation Oncologist	Tom Baker Cancer Centre
Dr.	Abhijit	Ghose	Radiation Oncologist	Alberta Health Services
Dr.	Sharlene	Gill	Medical Oncologist	BC Cancer
Dr.	Kamal	Haider	MD	Saskatoon Cancer Centre
Dr.	Edward	Hardy	Medical Oncologist	IHA/BC Cancer
Mrs.	Eva	Hernandez	Registered Nurse	Cancer Care Manitoba
Dr.	Michael	Humphreys	Medical Oncologist	BC Cancer
Dr.	William	Hunter	Radiation Oncologist	CancerCare Manitoba
Dr.	Will	Jiang	Resident (R5)	Tom Baker Cancer Centre
Mrs.	Jessica	Kasnik	Dietitian	Cross Cancer Institute
Dr.	Marc	Kerba	Radiation Oncologist	Tom Baker Cancer Centre
Dr.	Christina	Kim	Medical Oncologist	CancerCare Manitoba
Dr.	Sheryl	Koski	Medical Oncologist	Cross Cancer Institute
Dr.	Marianne	Krahn	Medical Oncologist	CancerCare MB
Dr.	Duc	Le	Radiation Oncologist	Saskatoon Cancer Centre
Dr.	Michael	Lee	Medical Oncology Fellow	BC Cancer
Dr.	Richard	Lee-Ying	Medical Oncologist	Tom Baker Cancer Centre
Mrs.	Stephanie	Lelond	Clinical Nurse Specialist	CancerCare Manitoba
Dr.	Howard	Lim	Medical Oncologist	BC Cancer
Dr.	Hongwei	Liu	Radiation Oncologist	Central Alberta Cancer Center
Dr.	Shaun	Loewen	Radiation Oncologist	Tom Baker Cancer Centre
Dr.	Shazia	Mahmood	Radiation Oncologist	Saskatchewan Cancer Agency
Dr.	Simon	Mairs	Medical Oncologist	Alberta Health Services
Dr.	Karen	Mulder	Medical Oncologist	Cross Cancer Institute
Dr.	Kim	Paulson	Radiation Oncologist	University of Alberta
Ms.	Carla	Pires Amaro	Medical Oncology Fellow	Tom Baker Cancer Centre
Mrs.	Elvira	Planincic	Registered Nurse	Cancer Care Manitoba
Mrs.	Kimberly	Robins	Pharmacist	CancerCare Manitoba
Dr.	Diane	Severin	Radiation Oncologist	Cross Cancer Institute
Dr.	John	Shaw	HPB Surgeon	Royal University Hospital
Dr.	Rishi	Sinha	Radiation Oncologist	Tom Baker Cancer Centre
Ms.	Karen	Stolz	Registered Nurse	Cancer Care Manitoba
Dr.	Amina	Taleb	Medical Oncology Fellow	Tom Baker Cancer Centre
Dr.	Vincent	Tam	Medical Oncologist	Tom Baker Cancer Centre
Dr.	Keith	Tankel	Radiation Oncologist	Cross Cancer Institute
Ms.	Kathy	Trakalo	Research Nurse	CancerCare Manitoba
Dr.	Robin	Visser	Hepatobiliary Surgery	Health Sciences Center
Dr.	Ralph	Wong	Medical Oncologist	Cancercare Manitoba
Dr.	Nobby	Woo	General Surgeon	University of Manitoba
Dr.	David	Wu	Radiation Oncology R4	Tom Baker Cancer Centre
Dr.	Adnan	Zaidi	Medical Oncologist	Saskatchewan Cancer Agency
Dr.	Muhammad	Zulfiqar	Medical Oncologist	BC Cancer

**Table 2:** Breakdown of pancreatic enzyme replacement therapy components.

Enzyme	Lipase	Protease	Amylase
Cotazym ECS 8	10,800	45,000	42,000
Cotazym ECS 20	25,000	100,000	100,000
Creon 10	10,000	790	11,200
Creon 25	25,000	1600	25,500
Cotzym *	10,000	35,000	40,000
Viokace 20 880 *	20,880	112,500	113,400

* non-enteric PERT.

**Table 3:** Criteria for the Barcelona Clinic Liver Cancer (BCLC) staging system.

BCLC Stage	Tumor Stage	Child-Pugh Class	ECOG Performance Status
Very Early (0)	Single ≤ 2 cm	A	0
Early (A)	Single ≤ 5 cm Up to 3 lesions ≤ 3 cm	A, B	0
Intermediate (B)	Multifocal	A, B	0
Advanced (C)	Portal Vein Invasion Lymph Node Involvement M1 disease	A	1–2
End Stage (D)	Any	C	≥3

## Data Availability

Not applicable.
